# The relationship of the serum endocan level with the CHA_2_DS_2_-VASc score in patients with paroxysmal atrial fibrillation

**DOI:** 10.1186/s43044-021-00132-1

**Published:** 2021-01-14

**Authors:** Gökhan Ceyhun

**Affiliations:** Faculty of Medicine, Department of Cardiology, Ataturk University, Erzurum, Turkey

**Keywords:** Paroxysmal atrial fibrillation, Endocan, CHA_2_DS_2_-VASc

## Abstract

**Background:**

In this study considering the relationship between serum endocan and CHA_2_DS_2_-VASc score, we assumed that endocan level could be a new biomarker for stroke risk in patients with paroxysmal atrial fibrillation (PAF). It was examined that endocan could be an alternative to determine the risk of stroke and anticoagulation strategy in patients with PAF. The CHA_2_DS_2_-VASc scores were calculated for 192 patients with PAF, and their serum endocan levels were measured. The patients were divided into two groups as those with low to moderate (0-1) and those with high (≥ 2) CHA_2_DS_2_-VASc scores, and the endocan levels were compared between these two groups.

**Results:**

The serum endocan level was significantly higher in the high CHA_2_DS_2_-VASc score group (*p* < 0.001). In the multivariate logistic regression analysis, endocan, C-reactive protein, and low-density lipoprotein were found to be independent determinants of the CHA_2_DS_2_-VASc score. The predictive value of endocan was analyzed using the ROC curve analysis, which revealed that endocan predicted a high stroke risk (CHA_2_DS_2_-VASc ≥ 2) at 82.5% sensitivity and 71.2% specificity at the cutoff value of 1.342.

**Conclusion:**

This study indicates that endocan is significantly associated with CHA_2_DS_2_-VASc score. We demonstrated that endocan could be a new biomarker for the prediction of a high stroke risk among patients diagnosed with PAF.

## Background

Atrial fibrillation (AF) may cause various thromboembolic complications due to the effect of thrombogenic risk factors. It has an increasing incidence with age. It can manifest in various ways; thus, different classifications have been made. The American Heart Association and American College of Cardiology divided AF into four groups as intermittent paroxysmal AF (PAF) which may terminate spontaneously up to seven or with treatment, persistent AF that does not end after 7 days, long-lasting AF, lasting more than 12 months, and permanent AF in which rhythm control cannot be achieved but the treatment continues to control the heart [1]. AF causes serious mortality and morbidity with thromboembolic complications. The risk of stroke is five times greater in patients with AF, and the cause of a stroke in 20% of patients is AF [2]. Many studies have revealed no difference between PAF and persistent and permanent AF in terms of stroke [3, 4]. CHA_2_DS_2_-VASc is the most commonly used scoring system to determine the risk of stroke and anticoagulant therapy to prevent cerebrovascular events [5]. Anticoagulation is often recommended if the CHA_2_DS_2_-VASc score is ≥ 2. Although the guidelines lower recommendation anticoagulation for 0-1 points, this does not mean that the risk of stroke is very low [6]. Taking into account the bleeding status, there is no clear biochemical marker for the determination of the stroke risk and anticoagulation treatment decision other than scoring methods.

Endocan, formerly called endothelial cell-specific molecule-1 (ESM-1), is a proteoglycan molecule secreted from the heart, skin, kidneys, digestive system, lungs, liver, brain, lymph nodes, and the vascular endothelium of the thyroid gland [7].

Endocan has been accepted as an indicator of neovascularization, angiogenesis, and endothelial cell activation [8]. Endocan shows its effects by triggering the production of proinflammatory cytokines and regulating microvascular permeability and leukocyte adhesion [9]. The prognostic value of endocan has been demonstrated in inflammatory disorders, tumor progression, sepsis, transplant rejection, hypertension, and chronic kidney disease [10]. Similarly, a positive correlation has been identified between endocan and risk factors of cardiovascular events, such as hypertension, diabetes mellitus, and coronary artery disease [11, 12]. Many of these risk factors associated with endocan constitute the CHA_2_DS_2_-VASc score. In this regard, it is also possible to establish a close relationship between AF and endocan. When the literature is considered, this close relationship between CHA_2_DS_2_-VASc score and endocan level was not fully investigated. In this study, in order to determine the extent of this relationship, the serum endocan levels of patients with PAF were examined in relation to their CHA_2_DS_2_-VASc scores used to predict thromboembolic risk.

## Methods

This study included a total of 192 patients presenting to our clinic with acute PAF, for whom rhythm control was achieved and a PAF attack was detected in the cardiac event recorder within 10 days but no electrophysiological procedure was performed. The CHA_2_DS_2_-VASc score was calculated for each patient as follows: + 1 point for congestive heart failure, + 1 point for hypertension, + 2 points for age ≥ 75 years, + 1 point for diabetes mellitus, + 2 points for a previous stroke/transient ischemic attack, + 1 point for vascular disease, + 1 point for age between 65 and 74 years, and + 1 point for female gender [5]. The patients with a CHA_2_DS_2_-VASc score of ≥ 2 were considered to have a high risk of stroke and evaluated in one group and those with a score of 0 and 1 were considered to have a low and moderate stroke risk, respectively, and evaluated in another group. Malignant diseases, history of acute myocardial infarction and cerebrovascular disease, renal disease, thyroid dysfunction, type 1 diabetes, pregnant women were excluded from the examination. High sensitivity C-reactive protein > 10 mg/dl and persistent or permanent AF subgroups were excluded as many comorbid conditions were accompanied by them.

Blood glucose, creatinine, complete blood count, serum lipid, total cholesterol, low-density lipoprotein cholesterol, high-density lipoprotein cholesterol (HDL-C), and triglyceride levels were measured from the blood samples taken from the antecubital vein after 12 h of fasting. For endocan, the blood samples taken from the same region were centrifuged at 2000 rpm for 15 min in a refrigerated centrifuge with a 30-min collection time. The separated serums were kept in a freezer at −80 °C. Twelve hours prior to laboratory analysis, the samples were allowed to defrost at + 4 °C. The samples were measured by enzyme-linked immunosorbent assay (ELISA) according to the manufacturer’s protocol (Shanghai Crystal Day Biotech Co Ltd., Shanghai, China), which has high sensitivity and specificity for detecting human endocan levels. The serum CRP levels (normal range 0-0.5) were measured by a nephelometric method (UniCel DxC 800 System, Beckman Coulter, Fullerton, CA, USA).

Statistical analyses were performed using the Statistical Package for the Social Sciences, version 20.0 (SPSS, Inc., Chicago, IL). The *t* test or the Mann-Whitney *U* test was used for continuous variables, and the data were expressed as mean ± standard deviation. Categorical variables were expressed as percentages and compared between the groups using the chi-square or Fisher’s exact test. The variables other than the components of the CHA_2_DS_2_-VASc score, which were found to be significant factors in the univariate analysis, were included in a multiple logistic regression analysis to determine the independent determinants of a high CHA_2_DS_2_-VAS score. A *p* value of < 0.05 was considered statistically significant. For the correlation analysis, Pearson’s and Spearman’s rank tests were conducted. Receiver operating characteristic (ROC) analyses were also undertaken to compare the performance power of endocan in determining the group with a high CHA_2_DS_2_-VASc score.

## Results

As a result of this study conducted with a total of 192 patients with PAF (mean age, 60.43 ± 3.22 years, 51% men), the mean CHA_2_DS_2_-VASc scores were calculated as 4.92 ± 2.77 for the group with a high stroke risk (CHA_2_DS_2_-VASc ≥ 2) and 0.19 ± 0.08 for the group with a low to moderate stroke risk. The baseline characteristics of all the patients are shown in Table 1. As expected, in addition to the CHA_2_DS_2_-VASc score components, the LDL, CRP, and endocan levels were higher for the patients at high risk of stroke while their white blood cell was statistically significantly lower.

**Table 1:** Baseline characteristics of the study population (*n* = 192)

*Variables*	*All patients* (*n* = 192)	CHA_2_DS_2_-VASc score ≥ 2 (*n* = 87)	CHA_2_DS_2_-VASc score 0-1 (*n* = 105)	*p value*
*Age, years*	60.43 ± 3.22	66.42 ± 11.4	55.47 ± 12.7	*< 0.001*
*BMI, kg/m*^*2*^	25.96 ± 1.04	26.01 ± 1.43	25.92 ± 0.94	0.248
*Hemoglobin, g/dL*	*13.35 ± 0.56*	*13.27 ± 0.72*	*13.41 ± 0.63*	*0.173*
White blood cell, 10^3^/mm3	*7.25 ± 2.76*	*6.13 ± 3.26*	*8.17 ± 2.07*	*0.002*
Platelet count, × 10^3^/μl	*278 ± 98*	*267 ± 78*	*303 ± 82*	*0.543*
*Creatinine, mg/dL*	*0.96 ± 0.05*	*1.01 ± 0.03*	*0.91 ± 0.04*	*0.491*
*Total cholesterol, mg/dL*	*188.52 ± 37.2*	*213.39 ± 45.7*	*167.92 ± 27.5*	*0.523*
*LDL-C, mg/dL*	*102.07 ± 14.3*	*127.7 ± 12.8*	*82.5 ± 14.2*	*0.004*
*Triglycerides, mg/dL*	*166.40 ± 31.2*	*168.5 ± 29.4*	*164.60 ± 36.2*	*0.372*
*Fasting glucose, mg/dL*	*103.2 ± 48.3*	*118.4 ± 37.3*	*90.5 ± 39.2*	*0.062*
C-Reactive protein (mg/dL)	*0.54* (0.26-0.87)	*0.83* (0.54-1.27)	*0.43* (0.26-0.87)	*< 0.001*
Serum albumin (g/dL)	3.68 ± 0.43	3.59 ± 0.28	3.77 ± 0.39	*0.423*
HbA1c (%)	*6.2 ± 0.3*	*6.4 ± 0.8*	*6.1 ± 0.9*	*0.003*
*CHA*_*2*_*DS*_*2*_*-VASc* (0-9)	*2.29 ± 1.46*	*4.92 ± 2.77*	*0.19 ± 0.08*	*< 0.001*
*Endocan, ng/mL*	1.249 ± 0.298	*1.776* ± 0.378	*0.812* ± 0.314	*< 0.001*
*Men, n* (%)	*98 (51.0)*	*43 (49.4)*	*55 (52.3)*	*0.002*
*Hyperlipidemic, n* (%)	*108 (56.3)*	*52 (59.7)*	*56 (53.3)*	*0.075*
*Diabetes, n* (%)	*133 (69.3)*	*67 (77.0)*	*66 (62.8)*	*< 0.001*
*Hypertension, n* (%)	*118 (61.5)*	*62 (71.2)*	*56 (53.3)*	*0.002*
*Smokers, n* (%)	*132 (68.8)*	*67 (77.0)*	*65 (61.9)*	*0.002*
*Hyperthyroidism, n* (%)	*19 (10)*	*9 (10.3)*	*10 (9.5)*	*0.873*
Previous medications, *n* (%)				
Aspirin	*82 (42.7)*	*43 (49.4)*	*39 (37.1)*	*0.062*
Other antiplatelet agents	*52 (27.0)*	*26 (29.8)*	*26 (24.7)*	*0.192*
ACEi/ARB	*65 (33.8)*	*30 (34.4)*	*35 (33.3)*	*0.532*
Beta-blocker	*45 (23.4)*	*24 (27.5)*	*21 (20)*	*0.112*
Calcium channel antagonist	*17 (8.8)*	*9 (10.3)*	*8 (7.6)*	*0.142*
Statin	*34 (15.6)*	*18 (20.6)*	*16 (15.2)*	*0.213*
Aldosterone antagonist	*51 (26.5)*	*25 (28.7)*	*26 (24.7)*	*0.052*
Verapamil/diltiazem	*18 (9.3)*	*11 (12.6)*	*7 (6.6)*	*0.512*
Diuretic	*6 (3.19)*	*2 (2.2)*	*4 (3.8)*	*0.031*
Antiarrhythmics (amiodarone, dronedarone, flecainide, propafenone, and sotalol)	*36 (18.7)*	*23 (26.4)*	*33 (31.4)*	*0.091*
VKA	*24 (12.5)*	*13 (14.9)*	*11 (10.4)*	*0.152*
NOAC	*58 (30.2)*	*52 (59.7)*	*6 (5.7)*	*< 0.001*

*BMI* Body mass index, *LDL* Low density-lipoprotein, *HbA1c* Glycosylated hemoglobin, *ACE* Angiotensin-converting enzyme inhibitor, *ARB* Angiotensin receptor blocker, *VKA* Vitamin K antagonist, *NOAC* Non-vitamin K antagonist oral anticoagulant

Using the univariate analysis, the variables showing with a high stroke risk positive or negative correlation were determined as white blood cell (*r* = −256, *p* = 0.002), LDL-C (*r* = 346, *p* < 0.001), CRP (*r* = 382, *p* < 0.001), and endocan (*r* = 301, *p* < 0.001). By excluding the CHA_2_DS_2_-VASc score components, the multivariate logistic regression analysis was conducted to identify the independent determinants of this score. LDL, CRP, and endocan (odds ratio, 1.862; 95% confidence interval, 1.358-4.064; *p* < 0.001) were found to be associated with a high CHA_2_DS_2_-VASc score after the potential confounding factors were adjusted (Table 2).

**Table 2:** Results of univariate and multivariate logistic regression analyses of a high CHA_2_DS_2_-VASc score (≥ 2)

	Univariate analysis			Multivariate analysis		
	Odds ratio	95% CI	*p* value	Odds ratio	95% CI	*p* value
LDL	1.634	1.028-2.248	0.002	1.132	1.173-2.091	0.042
CRP	1.956	1.813-5.099	< 0.001	1.196	1.013-2.699	0.017
Endocan	1.874	1.013-6.101	< 0.001	1.862	1.358-4.064	< 0.001

*CRP* C-reactive protein, *LDL* Low density-lipoprotein, *CI* Confidence interval

**Fig. 1:**
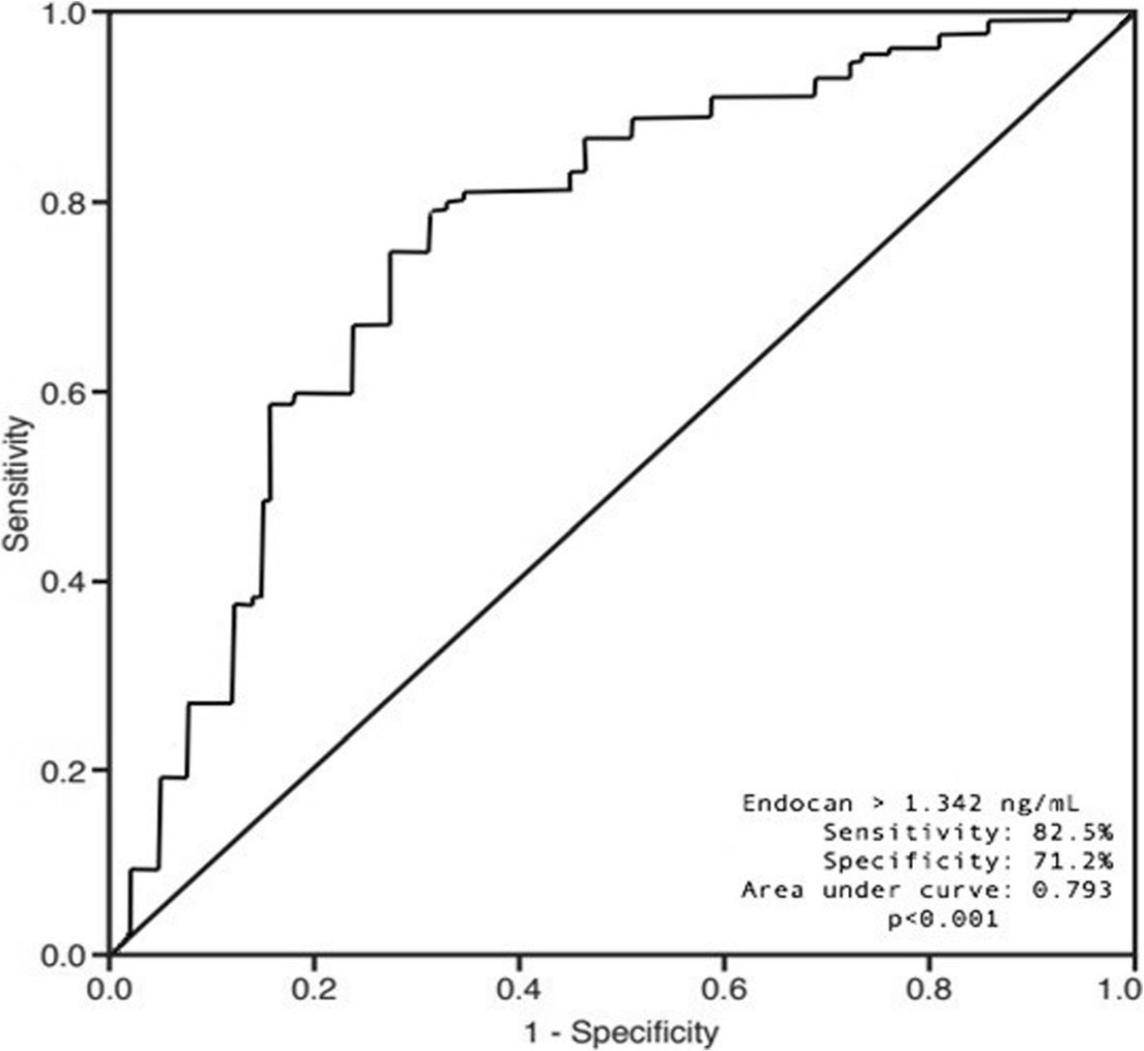
Area under the curve for endocan in determining a high CHA_2_DS_2_-VASc score (ROC curve)

ROC curves were used to determine the ability of endocan to predict high CHA_2_DS_2_-VASc scores. The area under the curve for endocan was 0.793 (95% CI 0.694-0.892; *p* < 0.001). Using a cutoff value of 1.342 ng/mL, endocan predicted high CHA_2_DS_2_-VASc scores at a sensitivity of 82.5% and specificity of 71.2% (Fig. 1). The risk of stroke was significantly lower in patients with endocan values lower than 1.342 ng/mL compared to the study population.

## Discussion

To the best of our knowledge, this is the first study to determine the close relationship between the serum endocan level and CHA_2_DS_2_-VASc score. Many studies have been conducted recently to investigate the relationship between cardiovascular diseases and endocan. It has been reported that in patients with acute myocardial infarction, an elevated level of endocan at the time of presentation to the hospital is an independent predictor of adverse cardiovascular outcomes, and it has also been independently associated with the severity of hypertensive coronary artery disease [13, 14]. Studies particularly focusing on the effects of cardiovascular diseases on the endothelium suggest that endocan may be a useful predictor of these diseases [15].

Risk factors, such as advancing age, hypertension, coronary heart disease, diabetes mellitus, valvular heart disease, heart failure, and hyperthyroidism cause structural changes in the heart resulting in the development of AF [16]. In fact, many conditions that cause vascular changes lead to the formation of AF, and several of these situations constitute the CHA_2_DS_2_-VASc score. On the other hand, many studies have shown that AF leads to endothelial dysfunction for many reasons, including increased shear stress, decreased nitric oxide bioavailability, increased oxidative stress, inflammation, and abnormalities of the renin-angiotensin system [17, 18]. As shown in a recently published meta-analysis, endocan released from endothelial cells increases in serum in disease that are components of the CHA_2_DS_2_-VASc score such as hypertension, diabetes, and coronary artery disease [19]. Similarly, we substantiated positive correlation between serum endocan level and CHA_2_DS_2_-VASc score in PAF patients.

Endothelial dysfunction mechanisms are closely related to the development of atrial cardiopathy [20]. It has been concluded that endothelial dysfunction, which is considered to develop as a result of AF, improves following the achievement of normal sinus rhythm [21, 22].

In addition, the detection of inflammatory infiltrates in the atrial and ventricular biopsies of patients with AF suggests that inflammation plays a role in AF [23]. Cox et al. reported that endocan played a significant role in endothelial dysfunction, since it regulated cell adhesion in inflammatory disorders [24]. Endothelial dysfunction and inflammatory damage mechanism caused by the components of the CHA_2_DS_2_-VASc score will lead to endocan release. Endothelial dysfunction, inflammatory damage seen in AF physiopathology, and indirect increased serum endocan level from components of the CHA_2_DS_2_-VASc score make it possible to observe a very close relationship between the CHA_2_DS_2_-VASc score and the endocan. In this study, a positive correlation was determined between endocan and the CHA_2_DS_2_-VASc score (≥ 2), an indicator of a high stroke risk, which includes a significant part of risk factors.

Chua et al. referred to the necessity of using biomarkers in AF and emphasized that markers, such as BNP and FGF-23 could be associated with AF [25]. There is not yet a biomarker predictive of thromboembolic complications. We determined a close relationship between CHA_2_DS_2_-VASc scores and endocan, which is an endothelium-specific marker. In addition, endocan was detected as an independent determinant of high CHA_2_DS_2_-VASc scores, which is an indicator of increased stroke risk in PAF in which many cardiovascular diseases may play a causative role.

The limitations of this study include the relatively small number of patients, observation nature of the study, and its lack of prospective validation. Also, the study is monocentric and does not comprise a large sample size.

## Conclusion

The results of our study clearly demonstrate that increased serum endocan in patients with PAF is a parameter that predicts a high risk of stroke. The use of endocan as a biomarker in patients with PAF will provide more objective evidence for a high stroke risk.

## Data Availability

All data and materials of the study are available.

## Abbreviations

PAF: Paroxysmal atrial fibrillation
AF: Atrial fibrillation
ESM-1: Endothelial cell-specific molecule-1
HDL-C: High-density lipoprotein cholesterol
